# Overcoming Trial Exclusion: A Multicenter Analysis of Hemodialysis Multiple Myeloma Patients Who Underwent BCMA-Directed CAR-T Therapy

**DOI:** 10.3390/curroncol33080475

**Published:** 2026-08-13

**Authors:** Eli Zolotov, Reema Mody, Kimberley Doucette, Aimee Chappell, Chantal Accolatse-Mati, Harsh Parmar, James Di Palma-Grisi, Noa Biran, Pooja Phull, Joseph Franz, David S. Siegel, David H. Vesole

**Affiliations:** 1 Division of Myeloma, John Theurer Cancer Center (JTCC), Hackensack University Medical Center, Hackensack, NJ 07601, USA; 2 Department of Medicine, Hackensack University Medical Center, Hackensack, NJ 07601, USA; 3 Department of Hematology and Medical Oncology, Medstar Georgetown University Hospital, Washington, DC 20007, USA

**Keywords:** multiple myeloma, CAR-T, BCMA, hemodialysis, end-stage renal disease, lymphodepletion, bendamustine, real-world evidence, health disparities

## Abstract

Multiple myeloma (MM) patients with end-stage renal disease (ESRD) requiring hemodialysis (HD), who are disproportionately Black, have been excluded from all pivotal trials of BCMA-directed chimeric antigen receptor T-cell (CAR-T) therapy, leaving a critical evidence gap in a population. We conducted a multicenter retrospective analysis of all MM patients who received CAR-T therapy while on HD at two U.S. centers between June 2021 and December 2025. Of 284 CAR-T recipients, 8 (2.8%) were HD-dependent. Median age was 63 years; 62.5% were female and 75% were Black; patients had received a median of 6 prior lines. Five received ciltacabtagene autoleucel and three received idecabtagene vicleucel; lymphodepletion was bendamustine (*n* = 4) or dose-reduced fludarabine/cyclophosphamide (*n* = 4). Cytokine release syndrome occurred in 37.5% (all Grade 1), with no grade ≥ 3 events—substantially lower than pivotal trials (76–95%). All 7 evaluable patients achieved neutrophil recovery by Day 30. One patient with prolonged pre-existing cytopenia pre-CAR-T therapy developed ICAN grade 4 and died from fungal pneumonia. Median progression-free survival was 17.5 months and median overall survival was not reached. Treatment-related mortality was 12.5%. CAR-T therapy is feasible, relatively safe, and effective in carefully selected HD-dependent MM patients, supporting expanded eligibility with appropriate dose modification.

## Simple Summary

Multiple myeloma (MM) patients with severe renal impairment, including those requiring hemodialysis (HD), were excluded from all major CAR-T therapy trials. As a result, data regarding the efficacy, safety, and feasibility of this treatment in HD-dependent patients, who are disproportionately represented among Black patients, remain limited. Our multicenter analysis included eight HD-dependent MM patients who underwent BCMA-directed CAR-T therapy. Treatment was generally well tolerated, with no cases of severe cytokine release syndrome and successful blood count recovery within the first month after treatment. The median progression-free survival was 17.5 months, while the median overall survival had not been reached. These outcomes compare favorably with those reported in the pivotal CAR-T trials from which these patients were excluded. No differences were observed between CAR-T products or lymphodepletion regimens. Our findings suggest that HD-dependent patients can safely benefit from CAR-T therapy and should not be routinely excluded from clinical trials.

## 1. Introduction

Multiple myeloma (MM) is the second most common hematologic malignancy, and renal impairment is among its most frequent and consequential complications. Approximately 50% of patients experience some degree of renal insufficiency during their disease course, and up to 16% progress to end-stage renal disease (ESRD), requiring hemodialysis (HD) [1,2]. The mechanisms of myeloma-related kidney injury are diverse, encompassing cast nephropathy, light chain deposition disease, AL amyloidosis, hypercalcemia, and drug nephrotoxicity. HD-dependent patients face substantially worse outcomes, with shorter survival and limited access to novel therapies [3].

These access barriers fall unevenly across populations. Black patients bear a disproportionate burden of MM, with an incidence more than double that of White patients, yet they comprise only up to 5% of participants in pivotal BCMA-directed chimeric antigen receptor T-cell (CAR-T) therapy trials [4,5]. Because Black patients have a higher prevalence of chronic kidney disease and ESRD, the renal function-based exclusion criteria common to these trials disproportionately affect them, and Black patients with myeloma are markedly less likely to receive CAR-T therapy than White patients [5,6]. The population most burdened by MM is thus left with the least evidence to guide treatment.

CAR-T therapy has transformed the treatment of relapsed/refractory MM (RRMM). Two commercially available products—idecabtagene vicleucel (ide-cel) and ciltacabtagene autoleucel (cilta-cel)—have produced deep and durable responses in pivotal trials [7,8,9,10]. However, patients with ESRD requiring HD were systematically excluded from these studies: KarMMa and KarMMa-3 mandated creatinine clearance (CrCl) ≥ 45 mL/min, and CARTITUDE-4 required CrCl ≥ 40 mL/min [7,8,9,10]. This exclusion creates a critical evidence gap for a vulnerable population with few therapeutic options.

A particular challenge in administering CAR-T therapy to HD patients is the selection and dosing of the lymphodepletion (LD) regimen. The standard fludarabine/cyclophosphamide (Flu/Cy) regimen requires modification because the active metabolite of fludarabine (2-fluoro-ara-A) is approximately 40% renally eliminated, and the U.S. Food and Drug Administration label recommends against its use at CrCl < 30 mL/min due to insufficient data [11]. Bendamustine, which is predominantly hepatically cleared, has emerged as a pharmacokinetically attractive alternative for LD in renal impairment [12,13]. However, no study has compared these two LD approaches in HD-dependent patients.

Published experience with CAR-T therapy in HD patients is limited to individual case reports of one to two patients [14,15]. Here, we present the largest multicenter analysis of BCMA-directed CAR-T therapy in HD-dependent MM patients, including the first comparison of bendamustine versus dose-reduced Flu/Cy (fludarabine and cyclophosphamide) lymphodepletion in this setting.

## 2. Materials and Methods

### 2.1. Study Design and Patient Selection

We conducted a multicenter retrospective analysis of all patients with MM who received CAR-T therapy at Hackensack University Medical Center and Medstar Georgetown University Hospital between June 2021 and December 2025. Patients were included if they required HD prior to and during CAR-T infusion. There were no exclusion criteria. Clinical follow-up was censored on 30 March 2026. The study was approved by the IRB of both participating institutions.

### 2.2. Data Collection

Data were extracted from electronic medical records and included patient demographics (age, sex, race, ethnicity), disease characteristics (myeloma subtype, cytogenetic risk, number of prior lines of therapy), CAR-T product type, lymphodepletion regimen and dosing, toxicity profiles, hematologic recovery parameters, and survival outcomes. Cytokine release syndrome (CRS) and immune effector cell-associated neurotoxicity syndrome (ICANS) were graded per the American Society for Transplantation and Cellular Therapy (ASTCT) consensus criteria [16]. Hematologic parameters (absolute neutrophil count [ANC], hemoglobin, and platelet count) were recorded at Day 30 and Day 90 post-CAR-T infusion.

### 2.3. Lymphodepletion Protocols

Two lymphodepletion regimens were used. The bendamustine regimen consisted of bendamustine administered at 90 mg/m^2^/day over 1–2 days. The Flu/Cy regimen consisted of a 3-day course with fludarabine off-label dose-reduction by approximately 50% from the standard 30 mg/m^2^/day (to approximately 15 mg/m^2^/day) owing to renal impairment, with cyclophosphamide at standard dosing (300 mg/m^2^/day). For patients receiving Flu/Cy, hemodialysis was performed 12 h after each dose of chemotherapy to facilitate drug clearance while allowing adequate exposure. The 12 h interval between fludarabine administration and hemodialysis was selected to balance two competing goals: (1) allowing sufficient time for intracellular uptake and phosphorylation of fludarabine to its active metabolite, which occurs within hours after infusion, and (2) initiating dialysis before excessive systemic accumulation occurs in the setting of impaired renal clearance.

### 2.4. Statistical Analysis

Descriptive statistics were reported as median with range for continuous variables and frequency with percentage for categorical variables. Progression-free survival (PFS) was defined as the time from CAR-T infusion to disease progression or death from any cause, whichever occurred first; patients alive without progression were censored at last follow-up. Overall survival (OS) was defined as the time from CAR-T infusion to death from any cause; surviving patients were censored at last follow-up. The Kaplan–Meier method was used to estimate PFS and OS. Given the small sample size, subgroup analyses were descriptive and exploratory. All subgroup analyses were considered exploratory and hypothesis-generating. Analyses were performed using Python (version 3.13; Python Software Foundation, Wilmington, DE, USA).

## 3. Results

### 3.1. Patient Characteristics

Among 284 patients who received BCMA-directed CAR-T therapy at the two participating institutions, 8 (2.8%) required maintenance HD and comprised the study cohort (Table 1). One patient (Patient 4) was excluded because he did not require HD prior to CAR-T treatment but only after. The median age at CAR-T infusion was 63 years (range 56–82). Most patients were female (62.5%), Black (75%), and non-Hispanic/Latino (87.5%). The predominant myeloma subtype was IgG (87.5%), with one patient having light chain-only disease. High-risk cytogenetics were documented in 2 of 5 evaluable patients (40%; 25% of the total cohort). The cohort was heavily pretreated, with a median of 6 prior lines of therapy (range 4–7).

### 3.2. Treatment Details

Five patients (62.5%) received cilta-cel and three (37.5%) received ide-cel. Lymphodepletion was evenly distributed, with four patients (50%) receiving bendamustine and four (50%) receiving Flu/Cy. Among bendamustine-treated patients, doses were provided as per their body surface area (90 mg/m^2^/dose), administered over 1–2 days. Among Flu/Cy-treated patients, fludarabine was dose-reduced by approximately 50% in all cases, with hemodialysis performed 12 h after each dose (Table 2). One facility provided only Flu/Cy to three of their patients due to shortage of Bendamustine. All patients remained dependent on HD throughout lymphodepletion and CAR-T therapy, and none recovered renal function to discontinue dialysis during follow-up.

### 3.3. Cytokine Release Syndrome and Neurotoxicity

The overall toxicity profile was favorable (Table 3). CRS occurred in 3 of 8 patients (37.5%), all Grade 1 (fever only). CRS was numerically more frequent with Flu/Cy (2/4, 50%) than bendamustine (1/4, 25%), and occurred exclusively in cilta-cel recipients (3/5, 60%) versus ide-cel (0/3, 0%).

ICANS occurred in 1 patient (12.5%), who experienced Grade 4 neurotoxicity following cilta-cel infusion with Flu/Cy lymphodepletion. This patient (Patient 9, age 78) had prolonged pre-existing fungal infection and cytopenias predating CAR-T therapy and died on Day 21 from invasive fungal pneumonia complicated by pulmonary hemorrhage in the ICU. A graphical comparison of CRS and ICANS rates by lymphodepletion regimen and versus pivotal trials is shown in Supplementary Figure S1.

### 3.4. Hematologic Recovery

Hematologic recovery data were available for 7 of 8 patients (Patient 9 died on Day 21 and was not evaluable). All 7 evaluable patients achieved neutrophil recovery (ANC ≥ 1000/µL) by Day 30 (Table 4). Median Day 30 ANC was 3960/µL (range 1000–7200) and median Day 30 platelet count was 114 × 10^3^/µL (range 36–288). At Day 90, median ANC was 3600/µL (range 1560–7250) and platelet counts ranged from 84 to 337 × 10^3^/µL.

When stratified by LD regimen, a trend toward higher Day 30 ANC was observed with bendamustine compared with Flu/Cy (median 4050 vs. 1720/µL). Day 30 platelet counts were also numerically higher with bendamustine (median 191 vs. 48 × 10^3^/µL), whereas Day 90 hemoglobin trended higher with Flu/Cy (median 11.4 vs. 8.8 g/dL). These numerical differences should be considered hypothesis-generating given the small sample sizes.

### 3.5. Infections and Mortality

Infections were documented in 2 of 8 patients (25%), both in the Flu/Cy group, and one patient required ICU admission (12.5%). Three deaths occurred during follow-up (37.5%): one from myeloma progression (Patient 5, at 3.7 months), one from an unrelated second malignancy (Patient 3, end-stage lung squamous cell carcinoma, at 13.6 months), and one treatment-related death (Patient 9, invasive fungal pneumonia with pulmonary hemorrhage, at 0.7 months). The treatment-related mortality rate was 12.5% (Table 5).

### 3.6. Survival Outcomes

**Figure 1:**
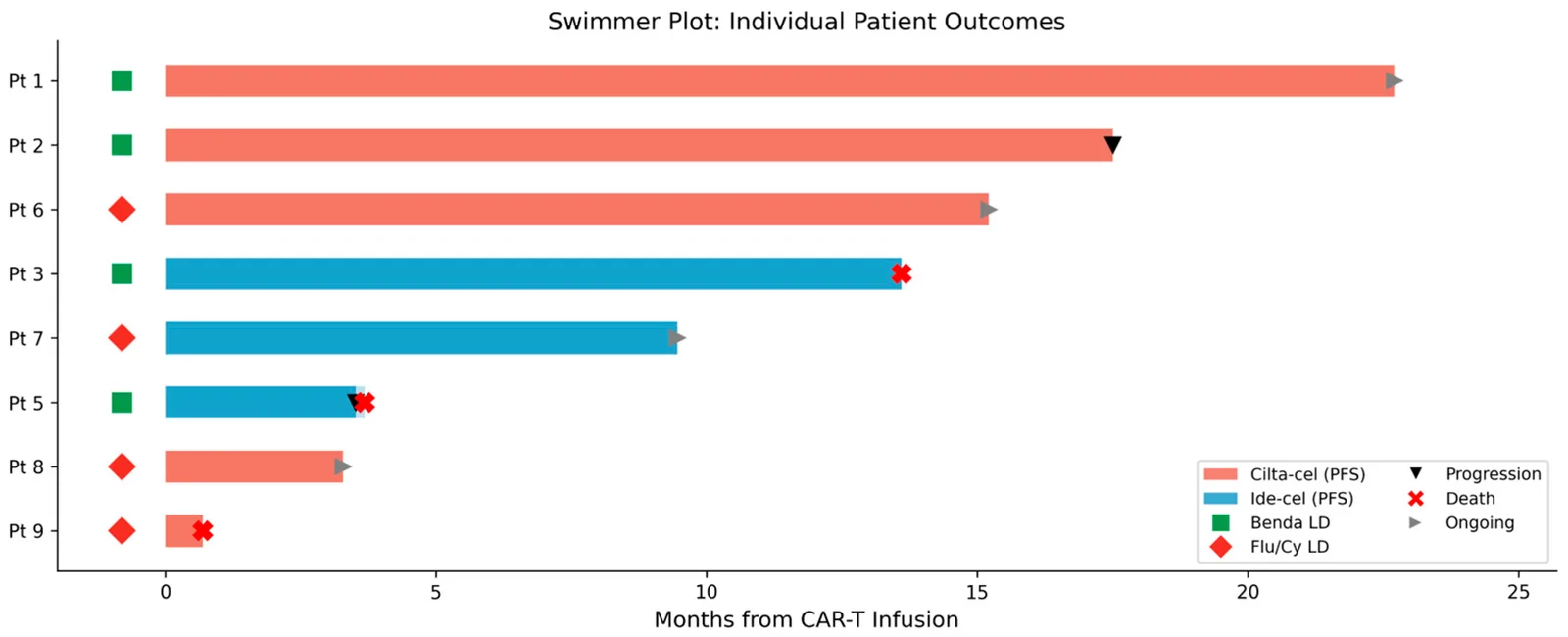
Swimmer plot of individual patient outcomes. Bar color indicates CAR-T product (red, ciltacabtagene autoleucel; blue, idecabtagene vicleucel). Left-side markers indicate lymphodepletion regimen (green square, bendamustine; red diamond, fludarabine/cyclophosphamide). Black triangle, progression; red X, death; arrow, censored/ongoing.

At a median follow-up of 11.5 months (range 0.7–22.7), the estimated median PFS was 17.5 months, and median OS was not reached (Supplementary Figure S2). Four PFS events occurred: two disease progressions, one death from an unrelated cause, and one treatment-related death. Among the 5 surviving patients, median follow-up was 15.2 months (range 3.3–22.7). Individual patient trajectories are depicted in the swimmer plot (Figure 1).

No apparent differences in PFS were observed between cilta-cel and ide-cel (median 17.5 vs. 13.6 months) or between bendamustine and Flu/Cy (median 13.6 months vs. not reached). Similarly, OS appeared comparable across the CAR-T product or LD regimen (Supplementary Figure S3).

## 4. Discussion

To our knowledge, this multicenter analysis of 8 HD-dependent patients represents the largest reported cohort of dialysis-dependent MM patients treated with CAR-T therapy. Our findings demonstrate that both ide-cel and cilta-cel can be administered safely and may be effective in carefully selected patients on maintenance HD, with encouraging survival and a favorable toxicity profile.

We showed low CRS incidence (37.5%, all Grade 1), substantially lower compared to pivotal KarMMa (84%), CARTITUDE-1 (95%), and CARTITUDE-4 (76%) trials [7,8,9,10]. Several mechanisms may contribute. First, although the role of HD in cytokine modulation remains uncertain, some degree of cytokine clearance (IL-1, IL-6, TNF-α), particularly with high cutoff membranes, has been reported, though conventional HD has shown limited and inconsistent removal of cytokines [17]. Second, the modified lymphodepletion intensity—through single-agent bendamustine or dose-reduced fludarabine—may attenuate CAR-T expansion and cytokine production. Third, the small sample size limits the precision of these estimates. Regardless of mechanism, the absence of severe CRS is reassuring and suggests carefully selected patients may be managed safely with appropriate monitoring.

This cohort is the first comparison of two LD strategies in HD-dependent patients. Bendamustine, with predominantly hepatic clearance, is an attractive alternative to Flu/Cy [12,13]. The trend toward higher Day 30 ANC and platelet counts with bendamustine aligns with reports in non-HD CAR-T populations [13]. Importantly, both strategies yielded comparable CRS/ICANS profiles and survival outcomes. The Flu/Cy approach requires careful attention to fludarabine dose reduction (~50%) and coordination with HD scheduling (12 h post-dose) but remains viable when bendamustine is unavailable; the choice between regimens may reasonably be guided by institutional preference and drug availability.

The efficacy outcomes compare favorably with pivotal trials. The estimated median PFS of 17.5 months exceeds KarMMa (8.8 months) and is comparable to KarMMa-3 (13.8 months), despite universal HD dependence and heavy pretreatment (median 6 prior lines) [7,8]. All 7 evaluable patients achieved neutrophil recovery by Day 30, and 12-month PFS and OS rates of 72.9% are encouraging. These outcomes suggest that renal function per se may not reliably predict CAR-T efficacy, consistent with a recent multicenter analysis showing comparable PFS and OS in patients with CrCl < 45 mL/min relative to those with preserved renal function [18,19].

One treatment-related death (Patient 9) deserves further discussion. This 78-year-old patient had months of pre-existing cytopenias and fungal infection predating CAR-T therapy and developed Grade 4 ICANS followed by fatal invasive fungal pneumonia on Day 21. This case underscores the importance of careful patient selection and highlights that pre-existing cytopenias in the context of ESRD, as well as uremic immune dysfunction, may compound the immunosuppressive effects of lymphodepletion and CAR-T-related hematotoxicity [18,19]. The CAR-HEMATOTOX score, which incorporates pre-LD hematologic and inflammatory parameters to predict prolonged cytopenias, may be a useful risk-stratification tool in this population [20].

Importantly, of these HD-dependent patients, 75% were Black, while in pivotal clinical trials there are fewer than 5% of them [4,5]. Racial disparities in treatment access and outcomes have been well documented in MM, including among patients receiving CAR-T therapy [21,22]. Our cohort shows how real-world demographics differ from the population registered to these trials. Because BCMA-directed CAR-T cells are not renally cleared, renal exclusion criteria principally reflect concerns about lymphodepletion toxicity rather than the cellular product itself. Our findings and emerging real-world data suggest these concerns can be mitigated through dose modification [12,18]. When access is provided with appropriate adaptation, outcomes appear comparable to the broader myeloma literature, and expanded eligibility, protocol-specified renal dose-modification arms, and diversity enrollment benchmarks are needed so that the benefits of CAR-T therapy reach all patients [23,24].

This study has several limitations. The small sample size (*N* = 8) limits statistical power and the precision of survival estimates. The retrospective design introduces potential selection bias and information heterogeneity, and the cohort is heterogeneous with respect to CAR-T products and LD regimens, limiting direct comparisons. We also lack a non-Black comparator group of HD CAR-T recipients and therefore cannot draw conclusions about racial differences in outcome; our findings speak to structural access rather than biology. Despite these limitations, the systematic exclusion of HD patients from clinical trials makes even small real-world cohorts a valuable addition to the evidence base.

## 5. Conclusions

BCMA-directed CAR-T therapy is feasible, relatively safe, and effective in carefully selected HD-dependent patients with RRMM. Both bendamustine and dose-reduced Flu/Cy are viable lymphodepletion strategies, with a signal favoring earlier hematologic recovery with bendamustine. These real-world data—drawn from a predominantly Black, doubly underrepresented population—suggest that HD-dependent patients may be considered for CAR-T treatment when appropriate dose modifications for renal impairment are implemented. Prospective studies and collaborative registry-based analyses are warranted to optimize management and validate these findings in larger cohorts.

## Figures and Tables

**Table 1:** Baseline demographics and disease characteristics.

Characteristic	All Patients (*N* = 8)
Age, years—median (range)	63 (56–82)
Female, *n* (%)	5 (62.5)
Male, *n* (%)	3 (37.5)
Black, *n* (%)	6 (75)
White, *n* (%)	1 (12.5)
IgG subtype, *n* (%)	7 (87.5)
Light chain-only, *n* (%)	1 (12.5)
Kappa/Lambda, *n* (%)	5 (62.5)/3 (37.5)
High-risk cytogenetics—Yes/No/Unknown, *n* (%)	2 (25%)/3 (37.5)/3 (37.5)
Prior lines—median (range)	6 (4–7)
Ciltacabtagene autoleucel, *n* (%)	5 (62.5)
Idecabtagene vicleucel, *n* (%)	3 (37.5)
Bendamustine LD, *n* (%)	4 (50)
Fludarabine/cyclophosphamide LD, *n* (%)	4 (50)

**Table 2:** Lymphodepletion regimens and dosing details.

Patient	Product	LD Regimen	Dose Details	HD Timing
1	Cilta-cel	Bendamustine	90 mg/m^2^/dose	Standard schedule
2	Cilta-cel	Bendamustine	90 mg/m^2^/dose	Standard schedule
3	Ide-cel	Bendamustine	90 mg/m^2^/dose	Standard schedule
5	Ide-cel	Bendamustine	90 mg/m^2^/dose	Standard schedule
6	Cilta-cel	Flu/Cy	Flu ~50% reduced + Cy standard × 3 days	12 h after each dose
7	Ide-cel	Flu/Cy	Flu ~50% reduced + Cy standard × 3 days	12 h after each dose
8	Cilta-cel	Flu/Cy	Flu ~50% reduced + Cy standard × 3 days	12 h after each dose
9	Cilta-cel	Flu/Cy	Flu ~50% reduced + Cy standard × 3 days	12 h after each dose

Flu, fludarabine; Cy, cyclophosphamide; HD, hemodialysis; LD, lymphodepletion.

**Table 3:** Toxicity profile by lymphodepletion regimen, *n* (%).

Toxicity	All (*N* = 8)	Benda (*n* = 4)	Flu/Cy (*n* = 4)
CRS	3 (37.5%)	1 (25%)	2 (50%)
ICANS	1 (12.5%)	0 (0%)	1 (25%)
Infection	2 (25%)	0 (0%)	2 (50%)
ICU admission	1 (12.5%)	0 (0%)	1 (25%)
Death (any cause)	3 (37.5%)	2 (50%)	1 (25%)
Treatment-related mortality	1 (12.5%)	0 (0%)	1 (25%)

Benda, bendamustine; Flu/Cy, fludarabine/cyclophosphamide; CRS, cytokine release syndrome; ICANS, immune effector cell-associated neurotoxicity syndrome.

**Table 4:** Hematologic recovery by lymphodepletion regimen.

Parameter (Median, Range)	Benda (*n* = 4)	Flu/Cy (*n* = 3)
ANC Day 30, /µL	4050 (3960–7200)	1720 (1000–2900)
ANC Day 90, /µL	4900 (1800–7250)	2500 (1560–3600)
Platelets Day 30, ×10^3^/µL	191 (103–288)	48 (36–114)
Platelets Day 90, ×10^3^/µL	164 (128–337)	95 (84–210)
Hemoglobin Day 30, g/dL	8.4 (8.2–9.9)	10.6 (8.2–10.6)
Hemoglobin Day 90, g/dL	8.8 (7.8–11.2)	11.4 (10.0–11.9)

ANC, absolute neutrophil count.

**Table 5:** Individual patient-level outcomes.

Pt	Age	Sex	Race	Product	Bridging Therapy /Response	LD	Prior Lines	CAR-HEMOATOTOX Score	CRS	ICANS	Infections	Best CAR-T Response	PFS, mo	OS, mo	Status/Cause of Death
1	61	F	Black	Cilta	-	Benda	6	-	0	0	No	CR	22.7	22.7	Alive
2	66	M	White	Cilta	Benda/Dex (PD)	Benda	7	-	1	0	No	CR	17.5	17.5	Alive
3	56	M	Black	Ide	Seli/Pom/Dex (PD)	Benda	7	-	0	0	No	VGPR	13.6	13.6	Dead, Lung SCC (unrelated)
5	60	M	Black	Ide	-	Benda	6	0	0	0	No	PR	3.7	3.7	Dead, Myeloma
6	67	F	Other	Cilta	Talq (VGPR)	Flu/Cy	5	-	1	0	No	CR	15.2	15.2	Alive
7	82	F	Black	Ide	-	Flu/Cy	4	2	0	0	No	VGPR	9.4	9.4	Alive
8	59	F	Black	Cilta	Car/Ven/Dex (-)	Flu/Cy	5	2	0	0	Yes	VGPR	3.3	3.3	Alive
9	78	F	Black	Cilta	Dara/Car/Dex (PD)	Flu/Cy	6	5	1	4	Yes	N/A	0.7	0.7	Dead, Fungal PNA

Cilta, ciltacabtagene autoleucel; Ide, idecabtagene vicleucel; Benda, bendamustine; Flu/Cy, fludarabine/cyclophosphamide; LD, lymphodepletion; CRS, cytokine release syndrome; ICANS, immune effector cell-associated neurotoxicity syndrome; Dex, dexamethasone; Dara, daratumumab; Car, carfilzomib; Pom, pomalidomide; Seli, selinexor; Talq, talquetamab; Ven, venetoclax; CR, complete response; VGPR, very good partial response; PR, partial response; PD, progressive disease; PFS, progression-free survival; OS, overall survival; SCC, squamous cell carcinoma; PNA, pneumonia; N/A, not applicable. Best response was assessed according to the International Myeloma Working Group (IMWG) response criteria.

## Data Availability

The de-identified data that support the findings of this study are available from the corresponding author upon reasonable request, subject to institutional data use agreements.

## Supplementary Materials

The following supporting information can be downloaded at: https://www.mdpi.com/article/10.3390/curroncol33080475/s1. Figure S1: Toxicity comparison. (A) CRS and ICANS incidence by lymphodepletion regimen. (B) CRS and ICANS incidence in the current study versus pivotal trials (KarMMa, CARTITUDE-1). Figure S2: Kaplan–Meier survival estimates (N = 8). (A) Progression-free survival; median 17.5 months. (B) Overall survival; median not reached. Figure S3: Kaplan–Meier estimates stratified by CAR-T product. (A) Progression-free survival. (B) Overall survival.
