# Supplementary material for: Overcoming Trial Exclusion: A Multicenter Analysis of Hemodialysis Multiple Myeloma Patients Who Underwent BCMA-Directed CAR-T Therapy
Source: Curr Oncol. 2026 Aug 13;33(8):475. doi: 10.3390/curroncol33080475 (PMC13510913; doi:10.3390/curroncol33080475)
Supplement: Supplementary file 1 [file curroncol-33-00475-s001.zip › curroncol-4433764-supplementary.pdf]

Supplementary Materials

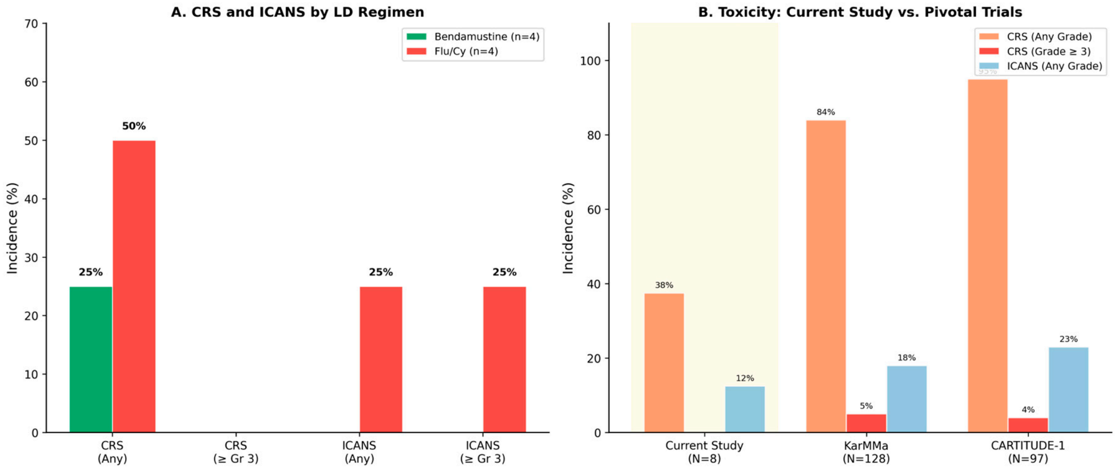

Figure S1. Toxicity comparison. (A) CRS and ICANS incidence by lymphodepletion regimen. (B) CRS and ICANS incidence in the current Study versus pivotal trials (KarMMa, CARTITUDE-1).

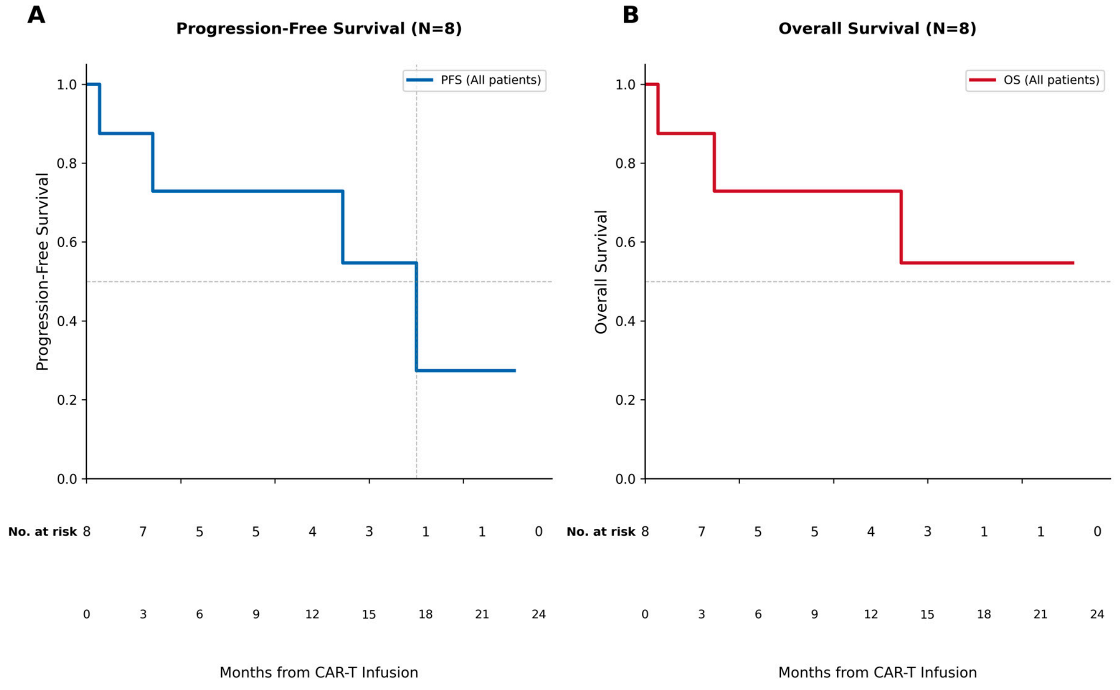

Figure S2. Kaplan-Meier survival estimates (N=8). (A) Progression-free survival; median 17.5 months. (B) Overall survival; median not reached.

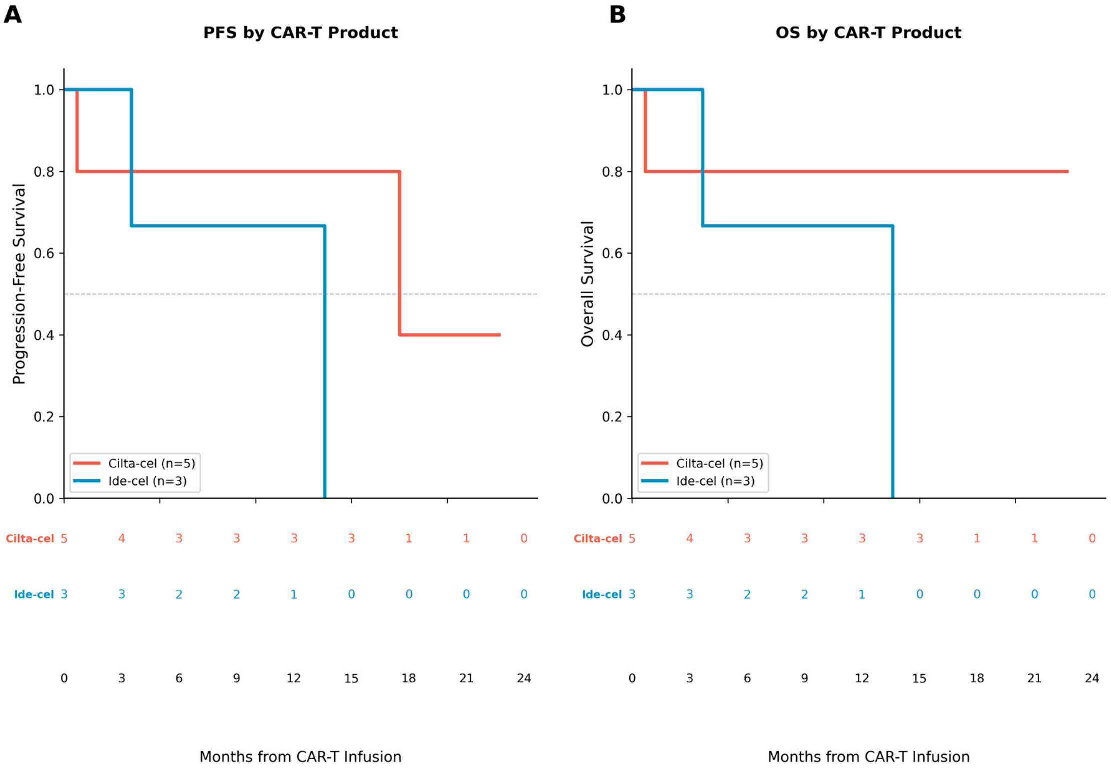

Figure S3. Kaplan-Meier estimates stratified by CAR-T product. (A) Progression-free survival. (B) Overall survival.
